# Adenovirus-mediated REIC/Dkk-3 gene therapy: Development of an autologous cancer vaccination therapy (Review)

**DOI:** 10.3892/ol.2013.1777

**Published:** 2013-12-27

**Authors:** MASAMI WATANABE, YASUTOMO NASU, HIROMI KUMON

**Affiliations:** 1 Center for Innovative Clinical Medicine, Okayama University Hospital, Okayama, Okayama 700-8558, Japan; 2 Department of Urology, Graduate School of Medicine, Dentistry and Pharmaceutical Sciences, Okayama University, Okayama, Okayama 700-8558, Japan

**Keywords:** REIC/Dkk-3, cancer vaccine, gene therapy, apoptosis, dendritic cells

## Abstract

Reduced expression in immortalized cells (REIC)/Dickkopf (Dkk)-3 is a tumor suppressor and therapeutic gene and has been studied with respect to the application of cancer gene therapy. Our previous studies demonstrated that the intratumoral injection of an adenovirus vector carrying the human REIC/Dkk-3 gene (Ad-REIC) suppresses tumor growth in mouse models of prostate, breast and testicular cancer and malignant mesothelioma. The mechanisms underlying these antitumor therapeutic effects have only been clarified recently. It has been demonstrated that Ad-REIC treatment inhibits cancer progression via the upregulation of systemic anticancer immunity. Under experimental conditions, autologous cancer vaccination via cancer-specific apoptosis and anticancer immune activation is a possible therapeutic mechanism. The robust anticancer effects observed in previous preclinical studies support the clinical utility of Ad-REIC. At present, a phase I–IIa study of Ad-REIC gene therapy in prostate cancer patients is ongoing. The current study reviews the observations of previous fundamental studies and summarizes the anticancer mechanisms of intratumoral Ad-REIC treatment in terms of cancer vaccination.

## 1. Introduction

A number of therapeutic cancer vaccines have been previously developed and evaluated in phase II/III clinical trials (1–3). The strategies for immunotherapy include the injection of peptides or proteins in adjuvant treatment and recombinant viruses and plasmids encoding immune factors, as well as the delivery of killed tumor cells and protein- or peptide-activated dendritic cells (DCs) to patients. With respect to the concept of cancer vaccination, controlling tumor-associated antigen (TAA) and systemic immune activation against TAA is essential. A number of previous clinical studies have been conducted focusing on single specific TAA molecules and designing a protocol to target TAA. However, previous trials of cancer vaccines have been unable to demonstrate robust therapeutic effects in spite of the activation of specific cytotoxic T lymphocytes against TAA (4). One possible reason for this is that targeting a single specific TAA is not sufficient to achieve substantial tumor reduction, since not all cancer cells express TAA and the cells without TAA escape the acquired immunity. In addition, there is a possibility that each immunological design of a cancer vaccine, such as specific peptides, is unlikely to cover the range of individual immune systems and may therefore, be ineffective. Hence, it is important to overcome the issues derived from the limited abilities of selected TAA molecules and differences in the immunological characteristics of patients.

A good strategy to address these issues is to kill the cancer cells at the tumor site via the direct injection of anticancer agents. In this way, the various TAAs released from the dead cancer cells are exposed to the individual immune system. The released TAAs are taken up by antigen-presenting cells (APC) and activated APCs upregulate the anticancer immune response by presenting the TAAs to immune effector cells (1,2). This type of therapy aims to use and vaccinate autologous tumors for anticancer immune activation. This autologous cancer vaccination strategy is predicted to activate individual immunity against the broad range of TAAs present in the cancer cells of the individual. This strategy is also attractive since systemic anticancer immunity may be activated simultaneously with substantial tumor reduction and these two therapeutic effects are predicted to be synergistic.

Gene therapy has been utilized in a number of previous clinical trials of human cancer and exhibits an innovative and attractive therapeutic potential. Adenovirus-mediated gene delivery continues to be the preferred treatment for cancer as the vectors indicate the high transduction efficacy of the therapeutic gene and the safety of the procedure when used for direct local injection (5–7). A number of previous clinical trials have demonstrated the utility and safety of the intratumoral injection of adenoviral vectors in cancer lesions (7). Adenoviral vectors are suitable for use in autologous cancer vaccination strategies as they succeed in robustly killing cancer cells and upregulating specific anticancer immunity pathways (6,8).

The reduced expression in immortalized cells (REIC) gene is identical to Dickkopf (DKK)-3 and REIC/Dkk-3 expression is significantly downregulated in a broad range of human cancer cells (9–17). In our previous study, the therapeutic effects of the REIC/Dkk-3 gene as a tumor suppressor gene were determined by the development of an adenovirus vector carrying the human REIC/Dkk-3 gene (Ad-REIC). The agent was found to significantly induce apoptosis in various cancer cells (13,18–20). Recently, the mechanisms of action of Ad-REIC agents in cancer gene therapy have been clarified *in vitro* and *in vivo* using several mouse tumor models. Under these experimental conditions, autologous cancer vaccination via cancer-specific apoptosis and anticancer immune upregulation is a potential therapeutic mechanism. We herein review the previously reported observations of fundamental studies and summarize the anticancer mechanisms of intratumoral Ad-REIC treatment in terms of cancer vaccination.

## 2. Characteristics of REIC/Dkk-3

The REIC gene was originally identified at Okayama University (Okayama, Japan) and reported in 2000 (9) as a gene whose expression is decreased via the immortalization of normal human fibroblasts. The authors performed mRNA expression profiling using subtractive hybridization of two types of cell lines, cobalt-irradiated normal fibroblasts, which stop proliferating and immortalized fibroblasts, which continue to proliferate. Subsequently, REIC was identified since expression of the gene was significantly reduced in the immortalized fibroblast cells. The sequence of the REIC gene was found to be consistent with that of the human Dkk-3 gene, a member of the Dkk family that encodes secreted proteins and consists of four primary members in vertebrates (Dkk-1, -2, -3 and -4). The expression of this gene was found to be markedly decreased in a variety of human immortalized cells and was therefore, named REIC. Previously, significant downregulation of the REIC/Dkk-3 expression has been reported in a broad range of human malignant tissues and REIC/Dkk-3 is hypothesized to function as a tumor suppressor gene (9–17).

The REIC/Dkk-3 gene is located on human chromosome 11p15.1 and contains 9 exons spanning >50 kbp (21). The REIC/Dkk-3 gene product is a secretory protein, while the gene itself encodes a deduced 38.3 kDa protein with 350 aa that is detected as two major bands of 60–68 kDa in size, according to variable glycosylation levels. The cDNA possess an N-terminal signal peptide, two cysteine-rich domains and two coiled-coil domains. REIC/Dkk-3 is an N-glycosylated protein, the majority of which intracellularly localizes to the endoplasmic reticulum (ER) (22). REIC/Dkk-3 is expressed in the majority of normal tissues in humans and mice, including the brain, heart, lungs, liver, colon and kidneys and is significantly downregulated in a broad range of human cancer cell lines (9,22). REIC/Dkk-3 has also been found to be downregulated in a variety of cancer tissues compared with surrounding normal tissue, including those of colorectal, lung, gastric, pancreatic, prostate, breast and bladder cancer, hepatocellular and renal cell carcinoma and malignant mesothelioma (10–17). Consistently, the REIC/Dkk-3 expression in cancer specimens is downregulated at the critical transition from low- to high-level malignant disease (10,13,16). Therefore, the lack of REIC/Dkk-3 expression has been found to positively correlate with the malignant grade and progression of cancer in several cancer types. Hypermethylation in the REIC/Dkk-3 promoter region has been previously reported in cancer cells with an absent or reduced expression (14,15,17,23).

## 3. Physiological functions of REIC/Dkk-3

Previously, the physiological functions of the REIC/Dkk-3 protein have been intensively investigated using knockout or overexpression of intracellular proteins. Previous studies have demonstrated that Dkk-3 modulates fibroblast growth factor and activin/nodal signaling to regulate the mesoderm induction of Xenopus. This suggests that physiological Dkk-3 is required for transforming growth factor β (TGF-β) signaling during early Xenopus development (24). Previously, the Dkk-3 protein has been found to also play an essential role in amphioxus head formation by inhibiting Wnt/β-catenin and nodal signaling (25). The authors identified that the Dkk-3 protein inhibits Wnt/β-catenin signaling in specific mammalian cells and cancer cell lines. However, Wnt/β-catenin signaling is positively regulated by the Dkk-3 protein in the murine retina and in several types of cell lines, including HEK293 cells (25). As for mammalian prostate glands, it has been previously reported that Dkk-3 is involved in prostate acinar morphogenesis and maintains the structural integrity of the prostate gland by limiting TGF-β/Smad signaling (26,27). Consistent with these observations, exogenous REIC/Dkk-3 protein promotes prostate acinar morphogenesis, suggesting that secreted REIC/Dkk-3 protein is also involved in prostate gland differentiation (22). In addition, the increased proliferation of human prostate epithelial cells has been previously confirmed in acini cells formed by epithelial cells stably silenced for Dkk-3 (27). Finally, the Dkk-3 gene has been found to be involved in the mechanisms underlying the differentiation of partially induced pluripotent stem cells to smooth muscle cells, thereby, transcriptionally regulating SM22 via the potentiation of Wnt signaling (28).

These results indicate that intracellular REIC/Dkk-3 protein plays a pivotal role in biology, involved in cell differentiation and proliferation and the development of specific organs via the regulation of the Wnt and TGF-β signaling pathways. Notably, REIC/Dkk-3 protein acts as an inhibitor or inducer of the Wnt and TGF-β signaling pathways based on the cellular conditions of various tissues and organisms, from amphioxus to vertebrates (25). The binding partner of intracellular REIC/Dkk-3 protein has been investigated by several previous studies. It has been reported that Dkk-3 binds to other proteins, such as Kremen1/2 (28,29), β-TrCP (30) and TcTex-1 (31), and that these interactions occur in the cytoplasm. These proteins include substantial molecules that significantly affect and modify intracellular signaling pathways, including the Wnt and TGF-β (16,32). Therefore, the functional varieties of intracellular Dkk-3 protein in the Wnt and TGF-β signaling may be partially explained by the various interaction partners or behavior of the proteins.

## 4. Cytokine-like aspects of exogenous REIC/Dkk-3 protein in monocyte differentiation

The immunological aspects of exogenous REIC/Dkk-3 protein have been investigated in a previous study (33). Purified recombinant proteins were added to human monocytes obtained from peripheral blood and the cytokine-like actions were examined. To clarify the effects of exogenous REIC/Dkk-3 protein on monocyte differentiation, human CD14^+^ monocytes were incubated with recombinant proteins at a concentration of 10 μg/ml. Recombinant REIC/Dkk-3 protein was found to induce monocyte differentiation to a DC phenotype. The morphological features of the REIC/Dkk-3-induced cell phenotype and its expression pattern of dendritic markers on the cell surface are similar to those of interleukin (IL)-4- and granulocyte macrophage-colony stimulating factor (GM-CSF)-induced DCs. Consistent with these observations, unpublished data indicates that recombinant REIC/Dkk-3 protein intraperitoneally administered in mice significantly upregulate the ratio of circulating DCs on flow cytometry. Recombinant REIC/Dkk-3 protein also possesses a cytokine-like function in the activation of STAT1 and STAT3 during dendritic phenotype differentiation *in vitro*. In terms of the expression of CD1a and CD14 surface markers, the REIC/Dkk-3-induced dendritic phenotype and IL-4- and GM-CSF-induced DCs are distinctly different. It is likely that these cells are categorized into DC subgroups. In addition, the direct cytotoxic effects of exogenous REIC/Dkk-3 protein were examined in several cancer cell lines. Even at a concentration of 20 μg/ml, no significant cytotoxic effects were associated with REIC/Dkk-3 protein treatment (33).

To date, the molecular mechanisms by which exogenous REIC/Dkk-3 protein differentiates monocytes into the DC phenotype have remained unclear. To clarify the immunomodulatory function of exogenous REIC/Dkk-3 protein, it is essential to determine the cell surface receptor. REIC/Dkk-3 is a secretory protein that is considered to act on cells via a cell surface receptor. However, the definitive cell surface receptors for this protein have not been identified. It has been previously reported that the expression levels of REIC/Dkk-3 modify intracellular Wnt and TGF-β signaling (24,25,27,29,30). There is a possibility that the REIC/Dkk-3 protein, secreted by the cells, interacts with unidentified cell surface receptors and exogenously affect these signaling pathways. Notably, several previous studies have demonstrated that Wnt and TGF-β signaling is involved in the differentiation of specific types of DC phenotypes (34–37). It is conceivable that modification of the signaling of the Wnt and TGF-β pathway by exogenous REIC/Dkk-3 protein triggers the differentiation to the DC phenotype in monocytes.

## 5. Adenovirus vectors expressing the human REIC/Dkk-3 gene (Ad-REIC) induce cancer cell-specific apoptosis

To examine the possible use of REIC/Dkk-3 as a tool for targeted gene-based therapy, we developed a replication-deficient adenovirus vector encoding the human REIC/Dkk-3 gene (Ad-REIC) (13). The CAG (CMV early enhancer/chicken β-actin) promoter was used to drive REIC/Dkk-3 expression, as this promoter enables strong gene expression (38,39). The overexpression of REIC/Dkk-3 induced by the Ad-REIC agent was found to stimulate apoptosis in a broad range of human cancer cell lines *in vitro* (13,18–20). By contrast, the ability of Ad-REIC to induce apoptosis was reduced in non-malignant cells (13,18,20). These observations indicate that the Ad-REIC agent selectively induces apoptosis in a cancer cell-specific manner. Since REIC/Dkk-3 expression is significantly downregulated in a broad range of human cancer cells, while being typically expressed in non-malignant or normal cells (9–17,22), the endogenous expression level of these proteins appear to correlate with sensitivity to the REIC/Dkk-3 overexpression induced by Ad-REIC.

**Figure 1:**
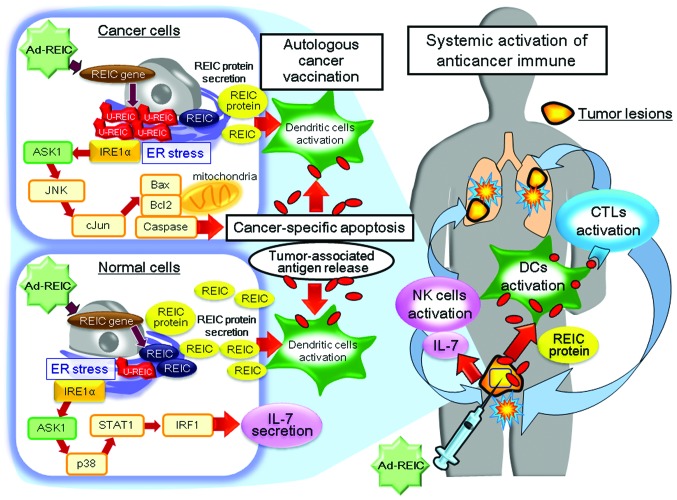
Therapeutic mechanisms of intratumoral Ad-REIC treatment. At the tumor site, Ad-REIC induces cancer cell-specific apoptosis in a phosphorylated JNK-dependent manner via ER stress signaling. Due to the poor capacity for the REIC/Dkk-3 gene expression, the cancer cells exhibit a failure to fold large amounts of REIC/Dkk-3 protein accumulated in the ER. This folding failure and the presence of U-REICs in the ER lead to the stress-induced apoptosis of cancer cells. The activation of JNK by Ad-REIC subsequently induces the phosphorylation of c-Jun, the translocation of Bax to the mitochondria and the downregulation of Bcl2, which subsequently leads to caspase-dependent apoptosis. By contrast, in non-cancer cells, which are typically resistant to Ad-REIC-induced apoptosis, a different ER stress response is observed following treatment. When REIC/Dkk-3 is overexpressed by Ad-REIC in normal cells, for example human fibroblasts, ER stress signaling of the p38, STAT1 and IRF1 pathways is activated. The activation of IRF1 then upregulates IL-7 expression and secretion in the cells. Autologous cancer vaccination with Ad-REIC treatment starts with cancer-specific apoptosis and the subsequent release of TAAs. The REIC/Dkk-3 protein, overexpressed and secreted by Ad-REIC transfection, differentiates monocytes into the DC phenotype at the tumor site. At the same time, abundant TAA fragments are released as a result of cancer cell-selective apoptosis and are supplied to the DCs induced by the secreted REIC/Dkk-3 protein. The activation of the DCs directly enhances cancer cell antigen presentation to CTLs, which upregulates systemic antitumor immunity. In addition, the enhanced IL-7 secretion observed in the normal cells activates NK cells, which also upregulates systemic anticancer immunity. The Ad-REIC-induced synergistic secretion of the REIC/Dkk-3 protein and IL-7 cytokines at the treated tumor site is important for the autologous cancer vaccination induced by the agent. These immunoactive proteins work together to mediate the phase of cancer-specific apoptosis to the anticancer immune effects observed at the injected and distant tumor sites. Ad, adenovirus; REIC, reduced expression in immortalized cells; JNK, c-Jun N-terminal kinase; ER, endoplasmic reticulum; Dkk-3, dickkopf-3; U-REICs, unfolded REIC/Dkk-3 protein; Bcl-2, B-cell lymphoma 2; Bax, Bcl-2-associated X protein; STAT1, signal transducer and activator of transcription 1; IRF1, interferon regulatory factor 1; IL, interleukin; TAAs, tumor-associated antigens; DC, dendritic cell; CTLs, cytotoxic T lymphocytes; NK, natural killer.

The molecular mechanisms underlying the apoptosis induced by Ad-REIC have been previously investigated and the pathway is shown in Fig. 1. The phosphorylation (activation) of c-Jun N-terminal kinase (JNK) is a critical step in cancer cell death (13,18,19). REIC/Dkk-3 protein is a secretory protein and the overexpression of this protein induced by Ad-REIC treatment efficiently leads to ER stress-induced apoptosis in cancer cells (19,21,40). ER stress-induced apoptosis is triggered due to a failure in the folding of large amounts of REIC/Dkk-3 protein accumulated in the lumen of the ER. The phosphorylation of JNK occurs downstream of ER stress signaling in cancer cells. As the REIC/Dkk-3 gene expression is absent or lacking in cancer cells (9–17,41), the REIC/Dkk-3 expression and protein folding system in cancer cells does not function well when the protein is overexpressed by Ad-REIC. This implies that, due to the poor capacity for REIC/Dkk-3 gene expression, the cancer cells easily exhibit a failure to fold large amounts of REIC/Dkk-3 protein accumulated in the ER. The differences in the capacity for REIC/Dkk-3 gene overexpression between cancer and normal cells may explain the cancer cell-specific apoptosis induced by Ad-REIC.

Previously, differences in ER stress signaling following Ad-REIC treatment have also been studied in cancer and normal cells (13,19,21,40). As shown in Fig. 1, REIC/Dkk-3-sensitive cancer cells, IRE1α (an ER stress sensor), apoptosis signal-regulating kinase 1 (ASK1) and JNK activation by Ad-REIC, subsequently induce the phosphorylation of c-Jun. As a result of c-Jun activation, translocation of Bcl-2-associated X protein to the mitochondria and the downregulation of B-cell lymphoma 2 occur, while the upregulation of caspases leads to the induction of apoptosis. By contrast, in non-cancer cells, which are typically resistant to Ad-REIC-induced apoptosis, a different ER stress response is observed following treatment. When the REIC/Dkk-3 gene is overexpressed by Ad-REIC in normal human fibroblasts, ER stress signaling with IRE1α, ASK1, p38, STAT1 and interferon regulatory factor 1 (IRF1) activation is observed, however, JNK activation is not detected. Furthermore, Ad-REIC treatment induces the upregulation of IL-7 expression and its significant secretion in human fibroblasts, based on IRF1 activation. These differences in the signaling of ER stress responses following REIC/Dkk-3 overexpression between cancer and normal cells may be involved in the various outcomes of apoptosis and the cancer cell-specific apoptosis induced by Ad-REIC agents.

## 6. Intratumoral Ad-REIC treatment robustly suppresses cancer growth in mouse tumor models

To demonstrate the therapeutic effects of Ad-REIC *in vivo*, mouse tumor models of several cancer types were established by transplanting the cancer cell line and treating the tumor-bearing mice with Ad-REIC (13,18–20,33,42,43). The tumor types of the model included prostate (13,33,42), breast (20), testicular (18) and gastric (43) cancer and malignant mesothelioma (19). Ad-REIC was administered intratumorally (prostate, breast and testicular cancer), intraperitoneally (gastric cancer) or intrapleurally (malignant mesothelioma) to inhibit the growth of cancer cells. In these *in vivo* experiments, significant inhibition of tumor growth was observed in the Ad-REIC-treated group, however, the tumors progressively grew in the control Ad-LacZ-treated groups. In specific experiments, the tumors that developed following Ad-REIC treatment were resected and examined using TUNEL staining to evaluate the induction of apoptosis by Ad-REIC. Significant numbers of TUNEL-positive cells were observed in broad areas of the Ad-REIC-treated tumors, however, few apoptotic cells were noted in the tumors of the control groups. In an orthotopic prostate tumor model established with a murine prostate cancer RM9 cell line, the progression of orthotopic tumor development and spontaneous metastasis to the retroperitoneal lymph nodes were robustly suppressed by intratumoral Ad-REIC administration (42). In addition, adenoviral vectors encoding the murine REIC/Dkk-3 gene similarly suppressed the progression of RM9 prostate cancer in the mouse model as well as the Ad-REIC encoding the human REIC/Dkk-3 gene. A series of *in vivo* experiments definitively indicated that the REIC/Dkk-3 gene is a promising molecule for cancer gene therapy and that the therapeutic utility of Ad-REIC agents may be applied in a broad range of human cancer types.

## 7. Adenovirus-mediated REIC/Dkk-3 gene therapy induces autologous cancer vaccination

Adenovirus-mediated REIC/Dkk-3 overexpression has been demonstrated to induce significant apoptosis in treated tumor sites and robust antitumor effects in mouse models (13,18–20,33,42,43). The induction of cancer cell-specific apoptosis by intratumoral Ad-REIC injection is an important therapeutic mechanism. When orthotopic RM9 prostate tumors are injected with Ad-REIC, significant apoptotic induction and tumor growth inhibition are observed in the treated lesions (33,42). Furthermore, treatment of orthotopic prostate tumors with Ad-REIC suppresses the tumor growth of distant lung metastasis in a mouse model (33). Previous *in vitro* cytolytic assays have demonstrated that anticancer immunity against RM9 prostate cancer cells is significantly enhanced in Ad-REIC-treated mice (33). These results indicate that intratumoral Ad-REIC injection in one tumor lesion also suppresses the growth of other distant cancer lesions via the induction of anticancer immunity. Primarily, intratumoral Ad-REIC treatment induces local apoptotic cell death in treated cancer sites and then activates systemic anticancer immunity against cancer cells. In addition, at the Ad-REIC-treated tumor site, secreted REIC/Dkk-3 protein plays a cytokine-like role in inducing monocyte differentiation to a specific DC phenotype and appear to be involved in systemic anticancer immunity (33).

As shown in Fig. 1, there are two mechanisms underlying the anticancer immune activation induced by Ad-REIC gene therapy. The first mechanism is based on autologous cancer vaccination, which is specifically observed in Ad-REIC gene therapy. Cancer vaccination with Ad-REIC starts with the cancer-specific apoptosis and subsequent release of TAAs. Since the REIC/Dkk-3 protein differentiates CD14^+^ monocytes into the DC phenotype (33), the REIC/Dkk-3 protein overexpressed and secreted by Ad-REIC transfection at the tumor site differentiates and activates DCs. At the same time, abundant TAA fragments are released as a result of cancer cell-selective apoptosis and supplied to the DCs induced by the secreted REIC/Dkk-3 protein. The activation of DCs directly enhances cancer cell antigen presentation to cytotoxic and helper T lymphocytes, which upregulate systemic antitumor immunity (1,2). Therefore, intratumoral Ad-REIC treatment activates the DCs via the actions of secreted REIC/Dkk-3 proteins and TAAs released at the Ad-REIC-injected tumor sites. Ad-REIC-based medicine is predicted to enhance systemic anticancer immunity and achieve antitumor effects in injected and distant lesions as a therapeutic cancer vaccine.

The second mechanism is based on the secretion of IL-7 observed in the normal stromal fibroblasts of the Ad-REIC-injected tumor sites (40). When the REIC/Dkk-3 gene is overexpressed by Ad-REIC in the fibroblasts of the treated tumor, significant levels of IL-7 are expressed and secreted in the cells. As shown in Fig. 1, this phenomenon is triggered by ER stress signaling via the actions of IRE1α and the activation of ASK1 and the p38 kinase system (40). The enhanced IL-7 expression and secretion observed following Ad-REIC treatment activate natural killer cells to play a role in the upregulation of systemic anticancer immunity (40,44). The Ad-REIC-induced synergistic secretion of REIC/Dkk-3 proteins and IL-7 cytokines at the treated tumor site is important for autologous cancer vaccination by the agent. Namely, these immunoactive proteins work together to mediate the phase of cancer-specific apoptosis to the anticancer immune effects observed at the injected and distant tumor sites. For these reasons, Ad-REIC-mediated medicine is predicted to provide autologous cancer vaccination therapy to be applied as an individualized tailor-made vaccine.

## 8. Future directions of Ad-REIC-mediated cancer vaccination therapy

The field of therapeutic cancer vaccination is currently undergoing a shift in focus, to individualize tailor-made vaccines and the targeting of multiple TAAs. Autologous tumor vaccines must be applicable vaccine formulations, as tumor cells are a clear source of TAAs for vaccination purposes and all relevant candidate TAAs must be contained within them (3). Ad-REIC-mediated cancer vaccination is based on the strategy of developing autologous cancer vaccines to achieve the individualized activation of antitumor immunity. The ultimate goal of Ad-REIC-mediated cancer vaccination is to cure not only the treated primary tumor, but also distant tumor lesions. Strategies of autologous cancer vaccination using intratumoral Ad-REIC treatment must also be available in the clinical setting.

Based on previous preclinical results and concepts, a phase I–IIa study of gene therapy using an Ad-REIC agent was initiated in January 2011 and is currently ongoing at Okayama University Hospital (Okayama, Japan). The aim of this clinical study is to verify the safety, efficacy and anticancer immunological effects of Ad-REIC-based gene therapy in prostate cancer patients. In particular, evidence of the concept of autologous cancer vaccination via Ad-REIC is being tested in clinical trials to clinically develop the agent for large-scale application. The safety and efficacy of Ad-REIC-mediated gene therapy have been verified in patients and the initial impression of the clinical trial has been good. We hypothesize that Ad-REIC-based medicine is likely to provide anticancer immunological effects via the application of autologous cancer vaccination, which is likely to become a promising therapeutic option for treating a wide range of human malignancies as a cancer vaccine.
